# The Gut Microbiota and Associated Metabolites Are Altered in Sleep Disorder of Children With Autism Spectrum Disorders

**DOI:** 10.3389/fpsyt.2020.00855

**Published:** 2020-09-02

**Authors:** Xueying Hua, Jiang Zhu, Ting Yang, Min Guo, Qiu Li, Jie Chen, Tingyu Li

**Affiliations:** ^1^ Children’s Nutrition Research Center, Children’s Hospital of Chongqing Medical University, Chongqing Key Laboratory of Childhood Nutrition and Health, Chongqing, China; ^2^ Department of Neonatology, Children's Hospital of Chongqing Medical University, Chongqing, China; ^3^ Ministry of Education Key Laboratory of Child Development and Disorders, National Clinical Research Center for Child Health and Disorders, China International Science and Technology Cooperation Base of Child Development and Critical Disorders, Chongqing, China

**Keywords:** gut microbiota, metabolism, autism spectrum disorders, sleep disorder, microbiota-gut-brain axis

## Abstract

**Background:**

Autism spectrum disorder (ASD) is a type of neurodevelopmental disease that is frequently accompanied by sleep disorder. Herein, we investigated changes in the gut microbiota and its metabolites correlated with core symptoms and sleep problems in children with ASD.

**Methods:**

One hundred and twenty children diagnosed with ASD based on Diagnostic and Statistical Manual of Mental Disorders (DSM-5) criteria were enrolled in our study. The Autism Behavior Checklist (ABC), Social Responsiveness Scale (SRS), and Childhood Autism Rating Scale (CARS) were used to assess autism symptoms, and the Children Sleep Habits Questionnaire (CSHQ) was employed to evaluate sleep problems in children with ASD. The 120 children were divided into a sleep disorder group (n = 60) and a control group without sleep disorder (n = 60) according to the CSHQ answers. Illumina MiSeq analysis of 16S rRNA genes was used to compare differences in gut microbiota, and metabolomics analysis was employed to asses associated metabolites.

**Results:**

SRS and CARS scores for the sleep disorder group were significantly higher than for the control group (*p* < 0.05). The abundances of butyrate-producing bacteria *Faecalibacterium* and *Agathobacter* were reduced significantly in the sleep disorder group (*p* < 0.05), and this was negatively correlated with CSHQ score (*p* = 0.007 and *p* = 0.014, respectively). The abundance of *Agathobacter* was also negatively associated with the ABC language score (*p* = 0.044). Furthermore, levels of 3-hydroxybutyric acid and melatonin were significantly lower (*p* < 0.05) while serotonin levels were higher (*p* < 0.05) in the sleep disorder group. The 3-hydroxybutyric acid level was positively associated with *Faecalibacterium* abundance (*p* = 0.000), and melatonin was positively associated with the abundance of *Faecalibacterium* (*p* = 0.036) and *Agathobacter* (*p* = 0.041). We also observed negative correlations between 3-hydroxybutyric acid and CSHQ (*p* = 0.000) and CARS (*p* = 0.009), between melatonin and CSHQ (*p* = 0.002) and ABC sensory score (*p* = 0.021), and a positive correlation between serotonin and CSHQ (*p* = 0.002) and ABC sensory score (*p* = 0.025).

**Conclusions:**

ASD children with sleep disorder exhibited declines in the abundance of *Faecalibacterium* and *Agathobacter*, decreased levels of 3-hydroxybutyric acid and melatonin, and an increase in serotonin. These changes may aggravate sleep problems and core symptoms in children with ASD.

## Introduction

Autism spectrum disorder (ASD) is a type of neurodevelopmental disease characterized by impaired social interactions and communication, accompanied by stereotypic behavior, and restricted interests or activities (1). In addition to these core symptoms, children with ASD often suffer from other developmental disorders, gastrointestinal symptoms, and sleep problems. Among these problems, several studies have shown that 50%−80% of children with ASD have sleep disorders (2–5). Common sleep problems include waking up at night, parasomnias, and sleep onset delay. Furthermore, the severity of core symptoms of ASD are higher in ASD children with sleep disorder (4).

Although ASD could be classified as a neurological disease in terms of pathology, there is increasing evidence to demonstrate that the gastrointestinal system and its resident microbiota plays an important role in certain symptoms of ASD. Changes in gut microbiota are common in children with ASD. Studies have reported a significant elevation in *Clostridium* spp., *Desulfovibrio* spp. and *Lactobacillus* in the intestines of children with ASD, and the ratio of *Firmicutes* to *Bacteroidetes* is lower than in normal controls (6–11). Most studies have shown that changes in the gut microbiota are closely related to the severity of autism (11, 12), anxiety, and depression (13). Social impairment was observed in germ-free mice (14), and probiotic therapy had a beneficial effect on autism behavior in autism animal models (15, 16). However, little research has been conducted on whether the gut microbiota can affect sleep problems in ASD.

Evidence suggests that brain function and behavior are affected by metabolites of the microbiota, and the key molecules are short-chain fatty acids (SCFAs) (17). As is common in gut dysbiosis, levels of SCFAs such as butyrate are decreased in ASD (18). Butyrate inhibits histone deacetylase (HDAC) enzymes which participate in the serotonergic system (19), and can induce melatonin (20). Elevated whole-blood serotonin levels were observed in patients with ASD following meta-analysis (21), and levels of melatonin in urine, plasma, and the pineal gland are also lower in patients with ASD (22). However, whether SCFAs such as butyrate produced by the gut microbiota are associated with sleep problems in children with ASD remains unknown.

Therefore, the present study aimed to investigate changes in the gut microbiota and associated metabolites in sleep problems, and explore correlations with core symptoms in children with ASD.

## Methods

### Patients

We collected 293 ASD children who completed the questionnaires, blood biochemistry test and collection of fecal samples in Hainan Province, China during 2018‑2019. And we divided these children into those with sleep problems (n=183) and no sleep problems (n=110) according to the standard of Children Sleep Habits Questionnaire (CSHQ), and 1:1 randomly selected 60 children with age and gender matching from each group.

Finally, a total of 120 children diagnosed with ASD, comprising 100 boys and 20 girls, were recruited in our study (60 suffered sleep disorder and 60 did not). Inclusion criteria were as follows: 1) all of these ASD children were diagnosed by a developmental pediatrician and psychologist in Hainan Women and Children’s Medical Center following a series of structured interviews according to the Diagnostic and Statistical Manual of Mental Disorders (DSM-5) criteria; 2) symptoms of patients were evaluated by the Autism Behaviour Checklist (ABC; score <53 is normal), the Childhood Autism Rating Scale (CARS; score <30 is normal) (23), and the Social Responsiveness Scale (SRS; score <65 is normal) (24); 3) sleeping conditions were evaluated by the Children Sleep Habits Questionnaire (CSHQ; score <41 is normal) (25); 4) parents of all ASD children signed written informed consent, and participation in this research was voluntary. Those with a history of other developmental disorders or psychiatric diseases, including genetic disorders with comorbid autism, Rett Syndrome, cerebral palsy, chronic seizures, other congenital diseases, and recent infection, were excluded.

The ethics review committee of the Institutional Review Board of Children’s Hospital, Chongqing Medical University, approved this study. This cross-sectional case-control study was based on a clinical trial which was registered in the Chinese Clinical Trial Registry (ChiCTR; registration number: ChiCTR-ROC-14005442).

### Questionnaire

Parents of ASD children completed the following questionnaires according to the standard instructions; a background questionnaire including questions concerning demographic data (e.g. name, age, gender) and medical history; a CSHQ comprising 41 questions, of which 33 were scoring items, divided into eight dimensions (bedtime resistance, sleep onset delay, sleep duration, sleep anxiety, night waking, parasomnia, sleep disordered breathing, daytime sleepiness). The CSHQ is an effective and reliable indicator that can effectively distinguish the sleep problems of clinical and community samples (e.g., Cronbach’s α = 0.68 for community samples and 0.78 for clinical samples) (26). Parents were asked to recall sleep behaviors occurring in a recent week. Items are rated on a 3-point scale; “usually” related to a sleep behavior occurring 5 to 7 times per week (3 points); “sometimes” related to 2 to 4 times per week (2 points); “rarely” related to 0 to 1 time per week (1 point). The higher the total score, the more serious the sleep problem. A total CSHQ score ≥41 was used as the criterion for evaluating sleep disorder (25).

### Stool Sample Collection

Fresh stool samples were collected from participants who did not receive antibiotic treatment and supplemental probiotics or prebiotics within the past month. Samples were placed in sterile stool tubes, frozen on dry ice within 24 h, and transported in a dry ice box to the Children’s Hospital of Chongqing Medical University within 5 days, where they were immediately stored at -80°C for future use.

### DNA Extraction and PCR Amplification

According to the manufacturer’s protocol, microbial DNA was extracted from stool samples using an OMEGA DNA Kit (Omega Bio-Tek, USA). The final concentration of purified DNA was determined using a NanoDrop 2000 UV-vis spectrophotometer (Thermo Scientific, Wilmington, USA), and the quality of DNA was checked by 1% agarose gel electrophoresis. Bacterial 16S rRNA gene V3−V4 hypervariable regions were amplified with primers 338F (5’-ACTCCTACGGGAGGCAGCAG-3’) and 806R (5’-GGACTACHVGGGTWTCTAAT-3’) using a GeneAmp 9700 PCR thermocycler system (ABI, USA). Thermal cycling included an initial denaturation at 95°C for 3 min, followed by 27 cycles 95°C for 30 s, annealing at 55°C for 30 s, elongation at 72°C for 45 s, and a final extension at 72°C for 10 min. Reactions were performed in triplicate 20 μl mixtures containing 4 μl of 5 × FastPfu Buffer, 2 μl of 2.5 mM dNTPs, 0.8 μl of each primer (5 μM), 0.4 μl of FastPfu Polymerase, and 10 ng of template DNA. The resulting PCR products were extracted from a 2% agarose gel, further purified with an AxyPrep DNA Gel Extraction Kit (Axygen Biosciences, Union City, CA, USA), and quantified with QuantiFluor-ST (Promega, USA) in accordance with the manufacturer’s protocol.

### Illumina MiSeq Sequencing

According to the standard protocols of Majorbio Bio-Pharm Technology Co. Ltd. (Shanghai, China), purified amplicons were pooled in equimolar concentrations and paired-end sequenced (2 × 300) on an Illumina MiSeq platform (Illumina, San Diego, USA). Raw reads were submitted to the NCBI Sequence Read Archive (SRA) database (Accession Number: SRA:254243).

### Processing of Sequencing Data

Raw fastq files were demultiplexed, quality-filtered by Trimmomatic, and merged by FLASH according to the following criteria: i) over a 50 bp sliding window, reads for any site with an average quality score <20 were truncated; ii) primers were exactly matched allowing two nucleotide mismatches, and reads containing ambiguous bases were deleted; iii) sequences with overlaps longer than 10 bp were merged in accordance with their overlapping sequence.

Operational taxonomic units (OTUs) were obtained with a 97% similarity cutoff using UPARSE (Version 7.1 http://drive5.com/uparse/) and chimeric sequences were identified and removed with UCHIME. The taxonomy of each 16S rRNA gene sequence was analyzed by the RDP Classifier algorithm (http://rdp.cme.msu.edu/) against the Silva (SSU132) 16S rRNA database using a confidence threshold of 70%.

### Microbial Analysis

OTUs with 97% similarity levels were analyzed for community richness (Chao, ACE, Sobs), community diversity (Shannon, Simpson), and rarefaction curve. Comparisons of Chao, ACE, Sobs, Shannon, and Simpson indices between sleep disorder and control groups were analyzed by student’s *t*-test. A partial least squares discriminant analysis (PLS-DA) was performed to effectively distinguish the groups.

### Metabolite Extraction

Stool samples (100 mg) were separately ground with liquid nitrogen and the homogenate was resuspended in prechilled methanol and 0.1% formic acid by vortexing thoroughly. Samples were incubated on ice for 5 min then centrifuged at 15,000 rpm at 4°C for 5 min. Some supernatants were diluted with liquid chromatography-mass spectrometry (LC-MS)-grade water to a final concentration of 60% methanol. Hereafter, samples were transferred into a fresh Eppendorf tube through a 0.22 μm filter then centrifuged at 15,000 g at 4°C for 10 min. Finally, the filtrate was injected into the LC-MS/MS system for analysis.

### UHPLC-MS/MS Analysis

LC-MS/MS analyses were performed using a Vanquish UHPLC system (Thermo Fisher) and an Orbitrap Q Exactive HF-X mass spectrometer (Thermo Fisher). Samples were injected onto an Hyperil Gold column (100 mm × 2.1 mm, 1.9 μm) at a flow rate of 0.2 ml/min and separated using a 16 min linear gradient. The eluents for positive polarity mode were eluent A (0.1% formic acid in water) and eluent B (methanol). The eluents for negative polarity mode were eluent A (5 mM ammonium acetate, pH 9.0) and eluent B (methanol). The solvent gradient was set as follows: 2% B 1.5 min, 2%−100% B 12.0 min, 100% B 14.0 min, 100%−2% B 14.1 min, 2% B 16 min. The Q Exactive HF-X mass spectrometer was operated in positive/negative polarity mode with a spray voltage of 3.2 kV, a sheath gas flow rate of 35 arb, an aux gas flow rate of 10 arb, and capillary temperature of 320°C.

### Metabolite Analysis

Compound Discoverer 3.0 (CD 3.0, Thermo Fisher) was used to process raw data files generated by UHPLC-MS/MS to perform peak alignment, peak selection, and quantification for each metabolite. The main parameters were set as follows: retention time tolerance 0.2 min, actual mass tolerance 5 ppm, signal intensity tolerance 30%, signal/noise ratio 3, minimum intensity 100,000. Peak intensities were normalized against the total spectral intensity, and normalized data were used to predict the molecular formula based on additive ions, molecular ion peaks, and fragment ions. Peaks were matched with mzCloud (https://www.mzcloud.org/) and ChemSpider (http://www.chemspider.com/) databases to obtain accurate qualitative and relative quantitative results.

### Statistical Analysis

Data were analyzed using SPSS statistical software (Version 17.0, SPSS Inc., USA) and GraphPad Prism (Version 5.0, GraphPad Software, San Diego California USA). Means, standard deviations (SDs), and percentages were used to describe all data, in addition to microbial data and metabolite levels. To compare levels between the two groups, two-tailed Student’s *t*-tests and the chi-square tests were used. To compare the abundances of microbiota and levels of metabolites between groups, two-tailed Student’s *t*-tests and the Mann-Whitney tests were used. To analyze the relationship between microbiota, metabolites, and scores for each scale, Spearman correlation analysis was used. Significance was presumed at *p <* 0.05.

## Results

A total of 120 children with ASD were recruited, and divided into a sleep disorder group (n = 60, 3.993 ± 0.1682 years old, 48 boys and 12 girls) and a no sleep disorder group (n = 60, 3.925 ± 0.1394 years old, 52 boys and 8 girls) according to the CSHQ score standard. As shown in **Table 1**, there was no significant difference in age and sex composition between the two groups (*p* > 0.05). The CSHQ score for the sleep disorder group was significantly higher than for the no sleep disorder group (45.50 ± 0.4002 vs. 36.52 ± 0.3457; *p* < 0.0001). Previous studies have reported that the severity of the core symptoms of ASD in ASD children suffering sleep problems is increased (4). Therefore, we compared ABC, SRS, and CARS scores among participants. As shown in **Table 1**, SRS and CARS scores for the sleep disorder group were significantly higher than for the no sleep disorder group (*p* < 0.05). These results suggest that core symptoms of autism are more severe in ASD children with sleep disorder.

**Table 1:** Comparison of general characteristics, CHSQ, and ASD Core Symptom Scale scores of ASD children with and without sleep disorder.

		No sleep disorder(n=60)	Sleep disorder(n=60)	*p-*value
**Age** (years), mean ± SD		3.925 ± 0.1394	3.993 ± 0.1682	0.7558
**Gender** (n)	**Male**	86.67% (52/60)	80.00% (48/60)	0.327
	**Female**	13.33% (8/60)	20.00% (12/60)	
**CHSQ**, mean ± SD		36.52 ± 0.3457	45.50 ± 0.4002	<0.0001***
**ABC**, mean ± SD		52.83 ± 3.258	57.27 ± 2.626	0.292
**SRS**, mean ± SD		87.48 ± 3.090	98.12 ± 2.442	0.008**
**CARS**, mean ± SD		35.90 ± 0.7432	38.28 ± 0.6592	0.018*

CHSQ, Children Sleep Habits Questionnaire; ABC, Autism Behavior Checklist; SRS, Social Responsiveness Scale; CARS, Childhood Autism Rating Scale; *p < 0.05, **p < 0.01, ***p < 0.001.

**Figure 1:**
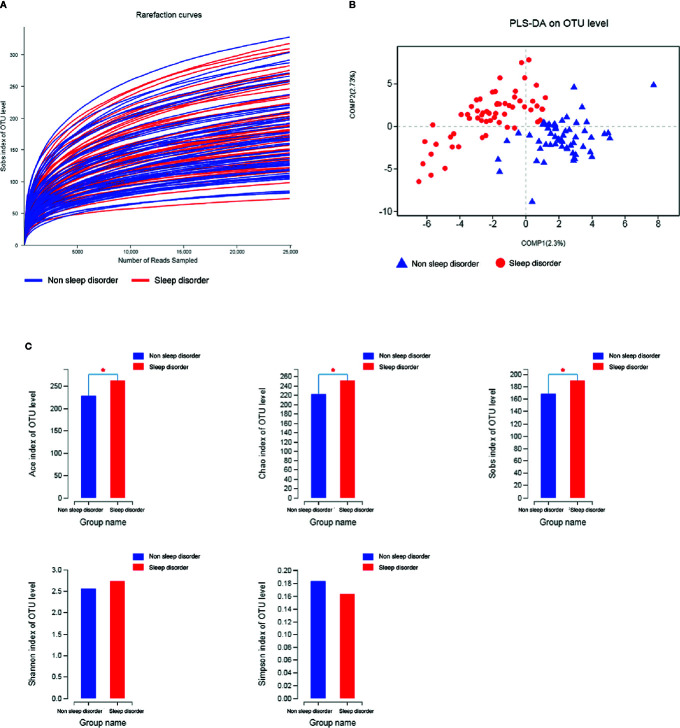
Rarefaction curves, PLS-DA, and α-diversity analysis of fecal samples from ASD children with and without sleep disorder. **(A)** Rarefaction curves calculated for OTUs with 97% identity for gut microbiota in sleep disorder and no sleep disorder groups. Blue curves represent the no sleep disorder group, and red curves represent the sleep disorder group. **(B)** PLS-DA plot based on unweighted UniFrac distance metrics. Blue triangles represent the no sleep disorder group, and red circles represent the sleep disorder group. **(C)** Comparison of ACE, Chao, Sobs, Shannon, and Simpson indices among the two groups. The blue column represents the no sleep disorder group, the red column represents the sleep disorder group, and α-diversity indices analyzed by Student’s *t*-test are reported as mean ± SD (*p < 0.05).

This study yielded 6,003,732 (50,031 reads per sample) high-quality sequences with an average length of 416.19 bp from 120 samples after OTU picking and chimera checking. We obtained numerous OTUs from valid sequences, and OTUs with 97% similarity were subjected to further statistical analysis. As the number of reads increased, the rarefaction curves of all samples shown in **Figure 1A** exhibited smooth growth trends and approached saturation plateaus, indicating that the amount of sequencing data obtained was sufficient.

An unweighted UniFrac distance matrix was calculated based on the OTUs of each sample to assess structural differences of microbial communities between the two groups. The results of the subsequent partial least squares discriminant analysis (PLS-DA) revealed obvious differences between the two groups (**Figure 1B**). This suggests that there may be differences in the distribution of the fecal microbiota between ASD children with or without sleep disorder.

The diversity indices ACE, Chao, and Sobs indicate the richness of the microbiota, whereas Shannon and Simpson diversity indices reflect the colony richness and evenness. As shown in **Figure 1C**, the ACE, Chao and Sobs diversity indices for the sleep disorder group were significantly higher than those for the no sleep disorder group (*p* < 0.05), while the Shannon index was higher and the Simpson index was lower for the sleep disorder group, but there were no significant differences between the two groups. These results demonstrated that the abundance and richness of the gut microbiota of ASD children with sleep disorder were greater than those of ASD children without sleep disorder.

**Figure 2:**
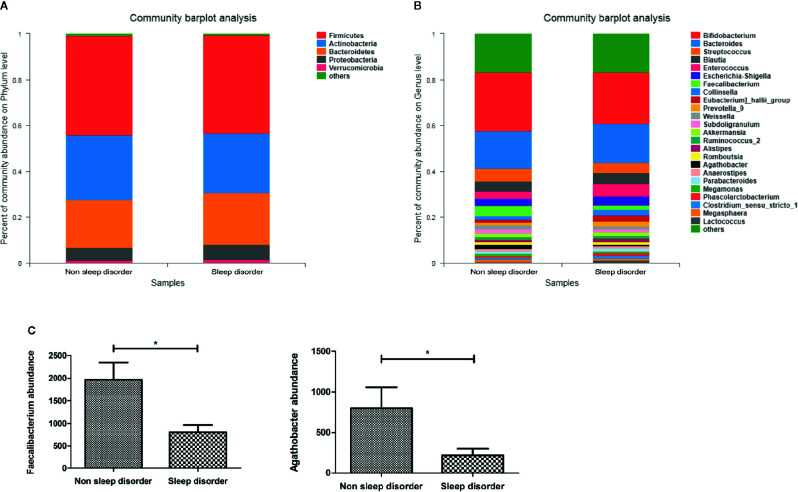
The relative abundance of bacteria in feces from ASD children with or without sleep disorder. **(A)** Dominant bacterial phyla with relative abundances greater than 1%. **(B)** Dominant bacterial genera with relative abundances greater than 1%. **(C)** Differences in *Faecalibacterium* and *Agathobacter* abundance in no sleep disorder and sleep disorder groups at the genus level (*p < 0.05, Mann-Whitney test).

**Figure 2A** shows the composition of the predominant microbiota in the two groups, and the relative abundance at the phylum level as >1%. In the sleep disorder group, the predominant phyla were *Firmicutes* (43.15%), *Actinobacteria* (25.88%), *Bacteroidetes* (22.57%), *Proteobacteria* (6.34%), and *Verrucomicrobia* (1.62%). Meanwhile, the predominant phyla were *Firmicutes* (43.32%), *Actinobacteria* (28.30%), *Bacteroidetes* (20.69%), *Proteobacteria* (5.58%), and *Verrucomicrobia* (1.29%) in the no sleep disorder group. However, there were no significant differences in these predominant phyla between the two groups (*p >* 0.05). Also, the *Firmicutes*/*Bacteroidetes* ratio was not significantly different (*p >* 0.05; data not shown). The composition of the predominant microbiota at the genus level is shown in **Figure 2B**, and the relative abundances in the two groups were >1%. As shown in **Figure 2C**, the abundances of *Faecalibacterium* and *Agathobacter* were significantly lower in the sleep disorder group than the no sleep disorder group (*p* < 0.05).

Furthermore, we also observed a significantly negative correlation between CSHQ scores and the abundances of *Faecalibacterium* (rs = -0.244, *p* = 0.007) and *Agathobacter* (rs = -0.225, *p* = 0.014) according to correlation analysis (**Table 2**). Additionally, the abundance of *Agathobacter* was negatively associated with the ABC language score (rs = -0.184, *p* = 0.044). These results suggest that the abundance of *Faecalibacterium* and *Agathobacter* might impact the severity of sleep disorder and autism core symptoms in ASD children. Several studies have reported that the gut microbiota may influence the central nervous system (CNS) by producing short-chain fatty acids (SCFAs) (27), and the main metabolite produced by *Faecalibacterium* and *Agathobacter* is butyrate, which belongs to the SCFAs.

**Table 2:** Correlation analysis between *Faecalibacterium* and *Agathobacter* abundance, and CHSQ and ASD core symptom score.

		*Faecalibacterium*		*Agathobacter*	
		rs	*p*-value	rs	*p*-value
**CHSQ**		-0.244	0.007**	-0.225	0.014*
**ABC**	**Total scores**	0.071	0.445	-0.008	0.931
	Sensory	-0.026	0.78	0.037	0.691
	Relating	0.083	0.368	-0.014	0.876
	Body and object use	0.097	0.292	0.022	0.812
	Language	0.069	0.457	-0.184	0.044*
	Social self-help	0.074	0.423	-0.047	0.607
**SRS**	**Total scores**	0.109	0.236	0.094	0.306
	Social awareness	0.131	0.155	0.059	0.523
	Social cognition	0.022	0.813	-0.026	0.78
	Social communication	0.164	0.074	0.084	0.363
	Social motivation	-0.015	0.867	0.133	0.149
	Autistic mannerisms	0.08	0.382	0.135	0.143
**CARS**	**Total scores**	-0.018	0.844	-0.046	0.616

CHSQ, Children Sleep Habits Questionnaire; ABC, Autism Behavior Checklist; SRS, Social Responsiveness Scale; CARS, Childhood Autism Rating Scale; *p < 0.05, **p < 0.01.

**Figure 3:**
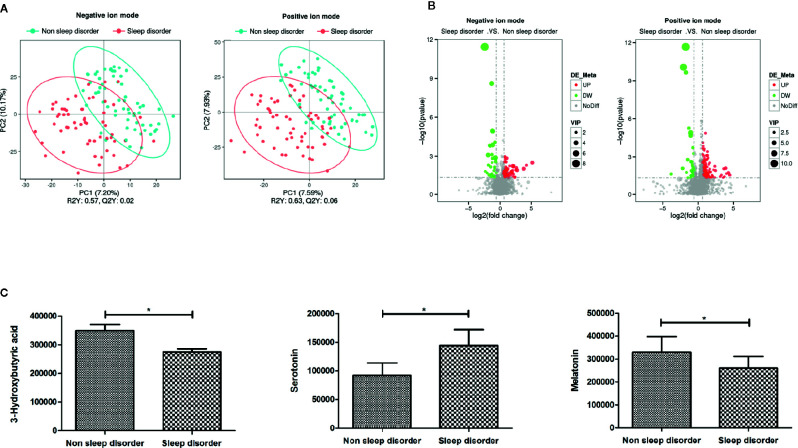
PLS-DA and differential metabolites of fecal samples from ASD children with and without sleep disorder. **(A)** PLS-DA analysis of metabolites using negative and positive ion modes. Blue circles represent the no sleep disorder group, and red circles represent the sleep disorder group. **(B)** Volcano maps of total differential metabolites in sleep disorder and no sleep disorder groups. Red dots represent elevated metabolites and green dots represent decreased metabolites. **(C)** The three main differential metabolites, 3-hydroxybutyric acid, serotonin, and melatonin, between sleep disorder and no sleep disorder groups (*p < 0.05, Student’s *t*-test and Mann-Whitney test).

To further explore the effect of the gut microbiota and its metabolites on the autism symptoms and sleep problems of children with ASD, we analyzed the metabolites of feces from the two groups. Analysis of metabolites in 120 fecal samples from ASD children with or without sleep disorder identified 4,531 species in positive ion mode and 2,405 species in negative ion mode. The results of subsequent PLS-DA are shown in **Figure 3A**, and there are clear differences between the two groups in both positive and negative ion mode. The total number of differential metabolites between the sleep disorder and no sleep disorder groups was 175 in positive ion mode and 99 in negative ion mode, and volcano maps are shown in **Figure 3B**. There were three main differential metabolites between sleep disorder and no sleep disorder groups (**Figure 3C**). Levels of 3-hydroxybutyric acid and melatonin were significantly lower in the sleep disorder group, while serotonin levels were significantly higher (*p* < 0.05). As shown in **Table 3**, 3-hydroxybutyric acid levels were positively correlated with *Faecalibacterium* abundance (rs = 0.382, *p* = 0.000), and the melatonin level was also positively correlated with both *Faecalibacterium* (rs = 0.197, *p* = 0.036) and *Agathobacter* (rs = 0.192, *p* = 0.041) abundance. Additionally, the 3-hydroxybutyric acid level was positively associated with melatonin level (rs = 0.782, *p* = 0.000; **Table 4**). The results suggest that there were changes in fecal metabolites related to key gut microbiota components in ASD Children suffering sleep problems, and the 3-hydroxybutyric acid level may influence the melatonin level, which is associated with sleep disorder (28–30).

**Table 3:** Correlation analysis between *Faecalibacterium* and *Agathobacter* abundance and metabolites levels.

	*Faecalibacterium*		*Agathobacter*	
	rs	*p*-value	rs	*p-*value
**3-Hydroxybutyric acid**	0.382	0.000**	0.054	0.582
**Serotonin**	0.097	0.294	0.103	0.262
**Melatonin**	0.197	0.036*	0.192	0.041*

*p < 0.05, **p < 0.01.

**Table 4:** Correlation analysis between serotonin, melatonin, and 3-hydroxybutyric acid.

	3-Hydroxybutyric acid	
	rs	*p-*value
**Serotonin**	0.054	0.558
**Melatonin**	0.782	0.000**

**p < 0.01.

Additionally, we analyzed the relationship between these three metabolites and CSHQ and autism core symptoms. We observed a negative correlation between 3-hydroxybutyric acid and CSHQ (rs = -0.441, *p* = 0.0001) and CARS scores (rs = -0.251, *p* = 0.009). There was a positive correlation between serotonin levels and CSHQ (rs = 0.276, *p* = 0.002) and ABC sensory score (rs = 0.205, *p* = 0.025). We also found that there was a negative association between melatonin level and CSHQ (rs = -0.284, *p* = 0.002) and sensory score in ABC (rs = -0.216, *p* = 0.021), as shown in **Table 5**. These results indicate that levels of 3-hydroxybutyric acid, serotonin, and melatonin may influence sleep and core symptoms in autistic children.

**Table 5:** Correlation analysis between serotonin, melatonin, 3-hydroxybutyric acid, CHSQ, and ASD core symptom score.

		3-Hydroxybutyric acid		Serotonin		Melatonin	
		rs	*p*-value	rs	*p-*value	rs	*p*-value
**CHSQ**		-0.441	0.000**	0.276	0.002**	-0.284	0.002**
**ABC**	**Total scores**	-0.112	0.255	0.069	0.457	-0.12	0.205
	Sensory	-0.179	0.067	0.205	0.025*	-0.216	0.021*
	Relating	-0.099	0.314	0.094	0.305	-0.031	0.741
	Body and object use	-0.028	0.775	0.006	0.952	-0.055	0.564
	Language	-0.054	0.585	0.015	0.868	-0.095	0.314
	Social self-help	-0.131	0.182	0.06	0.512	-0.14	0.139
**SRS**	**Total scores**	-0.093	0.341	0.119	0.197	-0.063	0.503
	Social awareness	-0.158	0.106	0.153	0.095	-0.172	0.067
	Social cognition	-0.125	0.203	0.107	0.247	-0.04	0.673
	Social communication	-0.036	0.716	0.144	0.118	-0.022	0.816
	Social motivation	-0.097	0.322	0.06	0.514	-0.113	0.231
	Autistic mannerisms	-0.071	0.469	0.051	0.581	-0.077	0.413
**CARS**	**Total scores**	-0.251	0.009**	0.076	0.406	-0.142	0.131

CHSQ, Children Sleep Habits Questionnaire; ABC, Autism Behavior Checklist; SRS, Social Responsiveness Scale; CARS, Childhood Autism Rating Scale; *p < 0.05, **p < 0.01.

## Discussion

Children’s sleep behavior and sleep quality are a comprehensive manifestation of psychological, developmental, biological, environmental, and cultural influences (31). Sleep disturbance may be harmful to children’s cognitive development and daily functions such as behavior, learning, attention, memory, and mood regulation (32, 33). Studies have reported that disordered sleep may be a contributing factor to problems in autism behavior (4, 34). In our present study, the core symptoms of ASD children also suffering sleep disorder were more severe than those experiencing normal sleep, consistent with previous studies.

Several studies have demonstrated changes in the gut microbiota of children with ASD compared with normally developing children (9, 12, 35, 36), and differences in microbial composition are correlated with alterations in behavior and cognition associated with the microbiota-gut-brain axis (37). Components of the microbiota communicate with the brain through various routes including the enteric nervous system, the immune system, tryptophan metabolism, and the vagus nerve, *via* several microbial metabolites such as SFCAs, peptidoglycan, and branched-chain amino acids (27). The microbiota-gut-brain axis may also be associated with the pathogenesis of ASD (38). In our current study, the results of PLS-DA showed that the distribution and composition of bacteria in feces from ASD children with and without sleep disorder were clearly distinct. Chao and ACE diversity indices reflect species richness of the microbiota, while Shannon and Simpson diversity indices indicate colony richness and evenness. In the present study, the abundance and richness of the gut microbiota in ASD children suffering sleep disorder were significantly greater than those of ASD children without sleep disorder. However, some previous studies on changes in gut microbial diversity have reported different results. Although the abundance and richness of the microbiota in the sleep disorder group were higher, changes in the billions of microbes within the microbiota in the human gut may vary greatly. In our study, we found that the abundance of two key bacteria, *Faecalibacterium* and *Agathobacter*, was significantly reduced in the sleep disorder group, at the genus level. Additionally, the abundance of *Faecalibacterium* and *Agathobacter* was negatively correlated with CSHQ scores, while the abundance of *Agathobacter* was also negatively associated with ABC language score. This indicates that the abundance of *Faecalibacterium* and *Agathobacter* may be associated with sleep problems and autism core symptoms.

*Faecalibacterium* is the most abundant bacterium in the human intestinal microbiota, accounting for more than 5% of the total number of bacteria present (39). *Faecalibacterium* is one of the most functionally active members of the microbiome (40), and one of the top butyrate-producing bacteria in the gastrointestinal tract (41). *Faecalibacterium* may affect physiological functions and homeostasis to maintain health by producing butyrate in the gut. *Agathobacter* species are anaerobic, Gram-positive bacteria that represent a novel species of a new genus in the family *Lachnospiraceae*. The main fermentation products of this genus are butyrate, acetate, hydrogen, and lactate (42). Butyrate is an SCFA that plays an important role in gut physiology. It has multiple effects on the intestinal cell life cycle, and many beneficial effects on health by preventing pathogen invasion, modulating the immune system, and reducing cancer progression (43). Butyrate is mainly produced by anaerobic microorganisms *via* the fermentation of indigestible carbohydrates through the acetyl-coenzyme A (AcCoA) pathway (17). Levels of butyrate are decreased in ASD patients (18).

The ketone body compound 3-hydroxybutyric acid can be synthesized from AcCoA by host cells, and it appears to interact with butyrate metabolism. Studies have reported that high levels of butyrate can raise the levels of 3-hydroxybutyric acid in calve blood and cerebrospinal fluid (CSF) (44). This indicates that 3-hydroxybutyric acid may be one of metabolites of butyrate. In the present study, we did not detect changes in butyrate level *via* analysis of fecal metabolites, but the 3-hydroxybutyric acid level was significantly lower in the sleep disorder group than the no sleep disorder group, and positively correlated with *Faecalibacterium* abundance. Previous studies also reported that butyrate directly affects the release of serotonin and intestinal hormones in the enteric nervous system, thereby stimulating the vagus nerve and eliciting endocrine signaling, both affecting brain function (17). In our present study, we found that serotonin were significantly elevated in ASD children suffering sleep disorder.

Serotonin is produced by intestinal chromaffin cells in the gut (45), and it plays a key role as a neurotransmitter in the brain by regulating various behavioral, autonomic, and cognitive functions (46). Studies have shown that compared with normally developing control groups, average serotonin levels in blood are significantly higher in ~30% of autistic individuals (47). Additionally, melatonin levels in urine, plasma, and the pineal gland can be lower in ASD individuals (20, 48–51). Melatonin is derived from serotonin and produced mainly in the pineal gland (50). Melatonin is a pleiotropic neuroendocrine molecule, which is essential for synchronizing circadian, sleep/wake cycles, and seasonal rhythms, and it also has antioxidant, neuroprotective, and immunomodulatory effects (28–30). In the present study, melatonin levels in feces of ASD children were significantly lower in the sleep disorder group than in the no sleep disorder group, and the abundance of *Faecalibacterium* and Agathobacter were positively associated with melatonin level, as were 3-hydroxybutyric acid levels, suggesting that butyrate-producing bacteria and butyrate-related metabolites were associated with melatonin. Furthermore, we found a negative correlation between 3-hydroxybutyric acid level and CSHQ and CARS. By contrast, there was a positive correlation between serotonin level and CSHQ and ABC sensory score. Additionally, the melatonin level was also negatively associated with CSHQ and ABC sensory score. This indicates that a reduction in butyrate and its related metabolites may affect serotonin levels, leading to a decrease in melatonin, which in turn may worsen sleep problems and core autism symptoms.

### Limitations

There are some limitations with the present work. This was a cross-sectional case-control study, and additional cohort studies should be performed to further explore the dynamic changes in sleep problems, symptoms, and gut microbiota in ASD individuals. A normal control group should also be added in future studies to compare differences in sleeping issues and gut microbiota between ASD and normal children. Furthermore, it remains unclear how gut microbiota may affect sleep conditions and autism symptoms, and the relationship between sleep problems and core symptoms also requires further investigation.

## Conclusions

In summary, sleep disorder is a common comorbidity in children with ASD that may exacerbate the core symptoms of ASD children. Thus, sleep problems have received widespread attention in recent years. Herein, the gut microbiota in ASD children with sleep problems was imbalanced, and the abundance of butyrate-producing bacteria *Faecalibacterium* and *Agathobacter* were reduced, leading to a decrease in butyrate and its metabolites, which further elicits an increase in serotonin and a decrease in melatonin, potentially aggravating sleep problems and core autism symptoms in children with ASD. Therefore, treatment strategies to ameliorate the gut microbiota and SCFAs, especially butyrate-producing bacteria, may have a potential role in relieving sleep disorder and core symptoms in ASD.

## Data Availability Statement

The datasets presented in this study can be found in online repositories. The names of the repository/repositories and accession number(s) can be found below: (https://www.ncbi.nlm.nih.gov/, SRA254243).

## Ethics Statement

The studies involving human participants were reviewed and approved by Institutional Review Board of Children’s Hospital, Chongqing Medical University. Written informed consent to participate in this study was provided by the participants’ legal guardian/next of kin.

## Author Contributions

XH and JZ conducted data collection and analysis, and drafted and revised the manuscript. JZ, TY, MG, and QL supervised the collection and analysis of ASD patient samples. TL and JC conceived and designed the research, performed data analysis and interpretation, revised the article, and conducted general supervision. All authors contributed to the article and approved the submitted version.

## Funding

This work was supported by the Key Scientific and Technological Projects of Guangdong Province (2018B030335001) and Guangzhou City (202007030002).

## Conflict of Interest

The authors declare that the research was conducted in the absence of any commercial or financial relationships that could be construed as a potential conflict of interest.
